# On the Correlation between Reservoir Metrics and Performance for Time Series Classification under the Influence of Synaptic Plasticity

**DOI:** 10.1371/journal.pone.0101792

**Published:** 2014-07-10

**Authors:** Joseph Chrol-Cannon, Yaochu Jin

**Affiliations:** Department of Computing, University of Surrey, Guildford, United Kingdom; SUNY Downstate MC, United States of America

## Abstract

Reservoir computing provides a simpler paradigm of training recurrent networks by initialising and adapting the recurrent connections separately to a supervised linear readout. This creates a problem, though. As the recurrent weights and topology are now separated from adapting to the task, there is a burden on the reservoir designer to construct an effective network that happens to produce state vectors that can be mapped linearly into the desired outputs. Guidance in forming a reservoir can be through the use of some established metrics which link a number of theoretical properties of the reservoir computing paradigm to quantitative measures that can be used to evaluate the effectiveness of a given design. We provide a comprehensive empirical study of four metrics; class separation, kernel quality, Lyapunov's exponent and spectral radius. These metrics are each compared over a number of repeated runs, for different reservoir computing set-ups that include three types of network topology and three mechanisms of weight adaptation through synaptic plasticity. Each combination of these methods is tested on two time-series classification problems. We find that the two metrics that correlate most strongly with the classification performance are Lyapunov's exponent and kernel quality. It is also evident in the comparisons that these two metrics both measure a similar property of the reservoir dynamics. We also find that class separation and spectral radius are both less reliable and less effective in predicting performance.

## Introduction

Reservoir computing has become a successfully applied recurrent neural network paradigm [1], [2]. It was initially introduced from both biologically inspired [3] and signal processing [4] groundings, and has since been applied successfully to real-world time-series pattern recognition problems [5], [6].

While the reservoir method has simplified the training of recurrent networks, the visibility into the workings of the internal computation remain largely opaque. In fact, we suggest that reservoir computing is more of a black-box than traditional feed-forward networks, because of the inability to trace clear paths from input features to internal nodes due to the highly recurrent connections.

The difficulty in functionally analysing reservoir networks has stifled attempts to improve the model parameters. Incorporating synaptic plasticity to adapt reservoir weights has been attempted [7], [8] and sometimes lead to improvements in performance [5], [6], [9]. However, the principles by which plasticity improves the parameters are not understood and reservoir adaptation is still essentially a trial and error affair. A recent review of computational models of neural plasticity and its role in self-organization of neural network models can be found in [10].

Some metrics for measuring reservoir characteristics have been put forward. These tend to center around the concepts of separation [11], edge-of-chaos criticality [12], and fading memory [13].

In this study, we will compare a selection of reservoir metrics on two time-series classification tasks, comparing the consistency between them. The stability of each metric will be studied by running each experiment over 10 random initialisations. A comparison will also be made of how three widely used plasticity rules and three initial connectivity structures affect each of the metrics. Finally, we look at the correlation between the metrics and classification accuracy to determine the extent that the metrics can be used to indicate performance.

These empirical comparisons will provide experimental guidance to complement the theoretical claims made for these measures.

## Results

The results are divided into two parts. The first part varies the reservoir connectivity and adaptation mechanism to show the effect this has on each of the metrics and classification performance. The second part shows how the metrics correlate with performance by plotting the quantities against each other and calculating Pearson's correlation.

### Effect of Plasticity and Connectivity on Metrics

Each of the figures in this section are box and whisker diagrams in which each box represents 10 randomly initialised simulation runs with a given parameter set indicated by the x-axis labels. The metric/performance is indicated on the y-axis.

#### Performance

**Figure 1:**
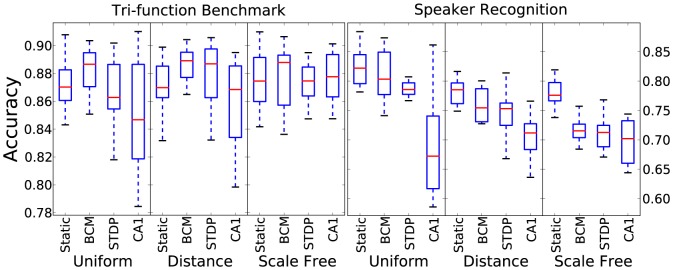
Classification accuracy results for 10 initialisations for each combination of plasticity rule, connectivity method and time-series task.

Figure 1 shows the results for performance, specifically the classification accuracy for each time-series task. In terms of reservoir adaptation, the Bienenstock, Cooper, Munro (BCM) rule produces slightly better results on the benchmark task while a static reservoir performs better on the speaker task. In both cases, tri-phasic STDP performs worse and has more variable results. For connectivity there is no significant trend, with uniform random connection performing somewhat better than the other two for the speaker task and somewhat worse for the benchmark.

#### Class separation

**Figure 2:**
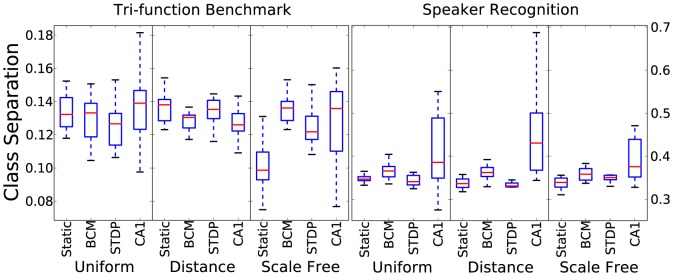
Class separation results for 10 initialisations for each combination of plasticity rule, connectivity method and time-series task.

Figure 2 shows the results for the class separation metric. Considering a higher class separation leads to better chances of learning, in theory, the tri-phasic STDP plasticity rule tends to give slightly better values for the metric. However, this form of plasticity is also the least stable and sensitive to initial connection/weight values, as can be seen by the significantly larger box size. The separation results vary drastically between the 2 time-series tasks tested. The speaker recognition task has much greater stability, indicated by smaller box size, apart from with the tri-phasic rule. Also interesting to note is that the class separation is higher on the speaker task than on the artificial benchmark data even though it has 9 classes compared with 3.

#### Kernel quality

**Figure 3:**
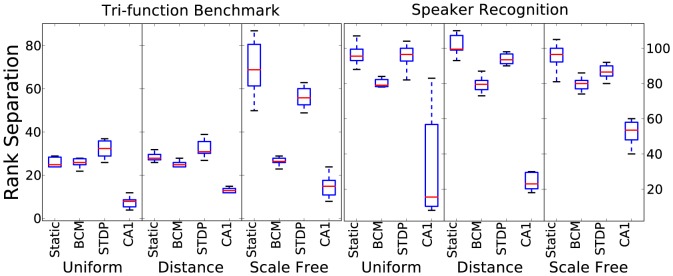
Kernel quality results for 10 initialisations for each combination of plasticity rule, connectivity method and time-series task.

Figure 3 shows the results for kernel quality. Kernel quality is also a measure to be maximised, with the greatest value in both tasks being 135, the dimension of the reservoir. Bi-phasic STDP and a static reservoir tend to give the best results for this measure. Tri-phasic STDP gives significantly lower, the opposite trend compared to class separation. Connectivity does not have a large effect, except scale-free producing better results for the benchmark task. The speaker recognition task again benefits from better values for this metric.

#### Lyapunov's exponent

**Figure 4:**
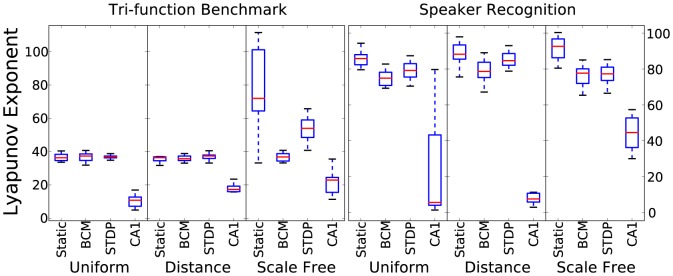
Lyapunov exponent estimate results for 10 initialisations for each combination of plasticity rule, connectivity method and time-series task.

Figure 4 shows the results for Lyapunov's exponent estimate. According to idea of desiring self-organised criticality, a value approaching 1, that represents the edge-of-chaos is ideal. Due to the dimension of the reservoir state, the results have been scaled by 135. Therefore 135 is the target value for these results. Strikingly, Lyapunov's exponent results follow kernel quality very closely. The relationship between them is almost exactly the same for the different reservoir settings which suggests that both play a similar role in estimating how rich the dynamics are in terms of computational transformation of the input.

#### Spectral radius

**Figure 5:**
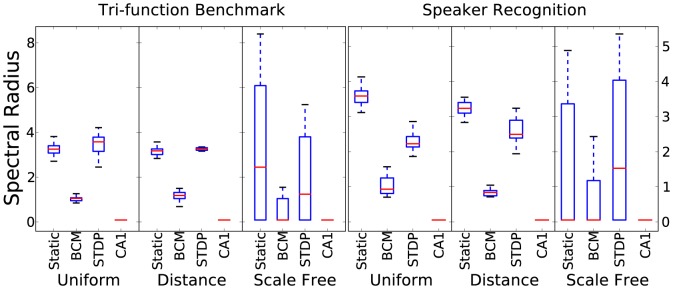
Spectral radius results for 10 initialisations for each combination of plasticity rule, connectivity method and time-series task.

Figure 5 shows the results for the spectral radius. Again, according to edge-of-chaos recurrent activity, this value is ideally approaching 1, at least claimed when dealing with non-spiking reservoirs [14]. Greater values than 1 will lead to instability of a supervised readout, while low values will lead to low computational power. The BCM rule consistently adapts the weights to give spectral radius values less than, but approaching 1. The other settings all lead to significantly higher values. Tri-phasic STDP always leads to weight matrices that are invalid for use with eigenvector detecting methods. This is also occasionally true with other plasticity rules when using scale-free connectivity. The numerical procedures to detect eigenvectors are approximate methods and not guaranteed to work with any arbitrary matrix.

### Metric Correlation to Performance

**Figure 6:**
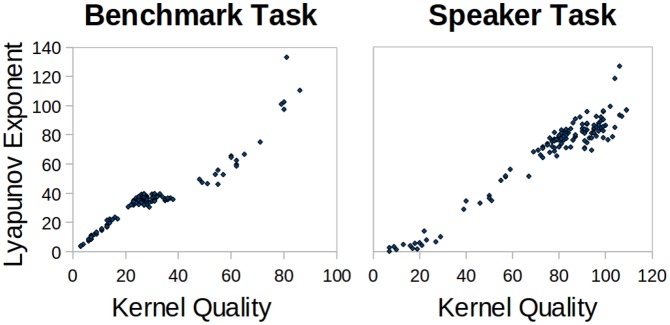
Lyapunov's exponent results plotted against kernel quality in both tasks to show the similarity between the metrics.

For all experimental simulation runs, the metric results are plotted against performance in Figure 6. This gives a visual indication of how strongly each metric can predict performance. For class separation, in both tasks there is practically no correlated pattern. Kernel quality and Lyapunov's exponent both show strong positive correlation for small values, but shortly level off and the pattern breaks down for large values. This could be due to the idea that it is only strictly necessary for the number of distinct reservoir states to exceed the number of input classes that require separation. This would explain why the 9-class speaker task has a shallower initial gradient than the 3-class benchmark. The spectral radius plots are distorted due to many failed calculations returning zero for the metric. Otherwise, there is significant negative correlation with the speaker task performance, but none for the benchmark.

To determine numerically how well each metric can be used to predict performance for a given reservoir, we look at Pearson's correlation results for each metric against both tasks, shown in Table 1. For each task there is a total of 120 reservoir initialisations from which the metric results are taken.

**Table 1:** Pearson's Correlation between Metrics and Performance.

Metric	PCC of Benchmark Task	PCC of Speech Task
Class Separation	−0.04	−0.2
Kernel Quality	0.22	0.29
Lyapunov's Exponent	**0.26**	**0.31**
Spectral Radius	0.05	−0.16

**Figure 7:**
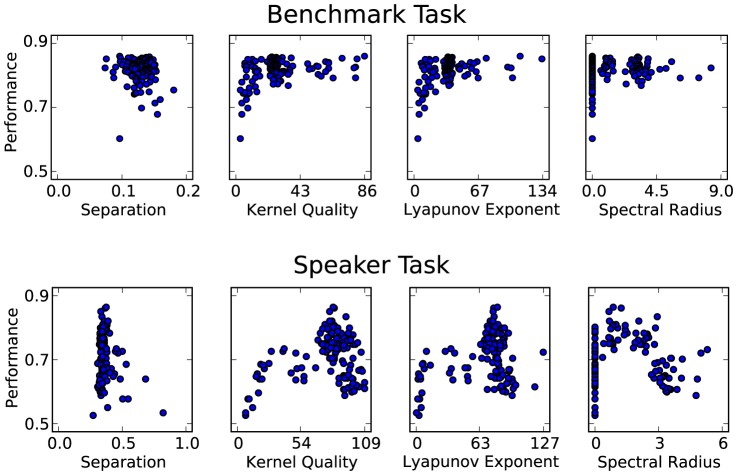
Each of the metrics for all simulation results plotted against classification accuracy in both tasks. This indicates the extent that each metric can be used to predict performance.

The two metrics that can give the strongest indication of performance in these tasks are Lyapunov's exponent followed closely by kernel quality. Their closeness in this aspect adds weight to the idea that they are measuring a similar property of a reservoir, in addition to the similar pattern of results in Figures 3 and 4. Figure 6 highlights the striking correlation between these metrics in both tasks. When visualising the link between Lyapunov's exponent and performance, the correlated trend is not as well defined. Although there is a significant correlation, Figure 7 shows that the metric has a large effect on performance only when it is within a small value range. As it increases, it seems to have less effect in determining performance.

For the benchmark task, class separation and spectral radius show no correlated pattern. Therefore, in this case, they do not give any hint to the performance at all. In the speaker recognition task, these metrics both show a significant negative correlation. However, this is not as strong as the positive correlation shown for Lyapunov's exponent and kernel quality. Surprisingly, class separation produces a negative correlation with performance where we would expect the opposite.

## Discussion

We have tested four of the most well established reservoir computing metrics on 2 classification tasks and under a number of different conditions. Out of these, 2 metrics have emerged as being more stable under a variety of settings: Lyapunov's exponent and kernel quality. Furthermore, both of these show remarkable similarity in the patterns they follow in their results. This leads to the conclusion that they are very likely measuring the same property of a reservoir. We suggest that this relates to the often used, but ill-defined phrase ‘rich reservoir dynamics’. In addition to their stability, it is these metrics that provide the best indication of performance, with Lyapunov's exponent coming out slightly ahead.

The spectral radius has a sole dependence on the weight matrix and is activity – and simulation – independent. Therefore, it would be highly beneficial for this measure to be utilised effectively, as it would indicate a reservoir's success before any simulation need commence. Unfortunately, in our case, the spectral radius does not provide a reliable indication of performance in the tasks we tested. Nor can it even be reliably computed, with tri-phasic STDP and scale-free connectivity producing weight matrices that were invalid for the metrics computational procedure.

Generally, tri-phasic STDP and scale-free connectivity led to worse values for each metric and a wider spread of results for each set of random initialisations. There are a couple of exceptions to this; 1) tri-phasic STDP produces higher class separation, 2) scale-free connectivity leads to higher kernel quality and Lyapunov's exponent for the benchmark task.

Class separation also fared poorly in that most set-ups gave a large spread within results for multiple initialisations. Also, it failed to reliably predict performance, giving no correlation for the benchmark task and fairly weak in the speech task.

## Materials and Methods

### Reservoir Network

**Figure 8:**
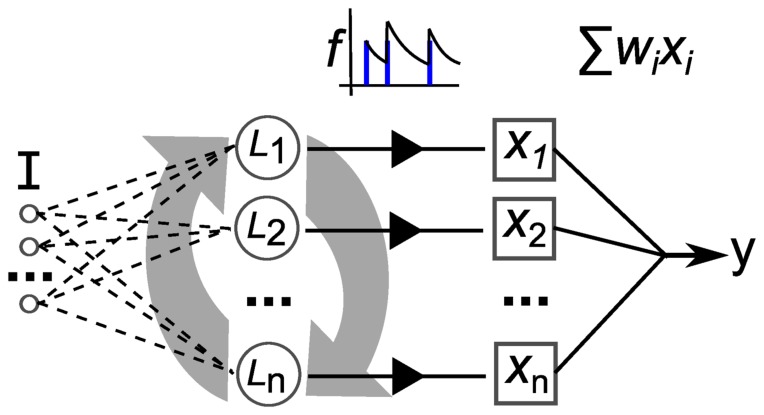
Depiction of the elements of our reservoir computing model. ***I*** is a multi-dimensional input signal, ***L*** nodes constitute the recurrent reservoir, the ***x*** vector is the reservoir state, ***f*** is the filtering of the spike trains and ***y*** is the output after weight and sum.

The reservoir model that we use is illustrated in Figure 8. The reservoir nodes, indicated by 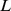 are stimulated by the inputs directly as injected current, 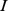, into the membrane potential modelled with Izhikevich's simple model [15]. The real-valued inputs are normalized between 0 and 1, which are multiplied by a scaling factor of 20 before being injected as current into 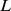. Input connections number 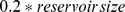, projected randomly to the reservoir nodes. The reservoir dynamics are then simulated for 150 ms. Then, the resulting spike trains produced by each of the reservoir nodes is passed through a low-pass filter, 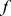, to produce a real valued vector used to train a linear readout.

Our reservoir consists of 135 spiking neurons with the ratio of excitatory to inhibitory as 4:1. Neurons are connected with static synapses (delta impulse function), according to connectivity described in the following subsection. weights are drawn from two Gaussian distributions; 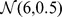 for excitatory and 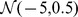 for inhibitory. When plasticity adapts the reservoir weights, 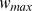 is clamped at 10 and 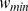 at -10. All parameters for excitatory and inhibitory neuron membranes are taken from [15].

To generate an output, the spike train from each reservoir node is low-pass filtered and a weight-and-sum readout is applied according to the methods in [3]. This output is trained with the iterative, stochastic gradient descent method: Least Mean Squares, given in Equation 1.
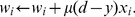
(1)


Here, 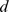 is the desired output, 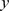 is the actual output, 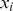 is the input taken from a neuron's filtered state, and 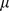 is a small learning rate. The weight from 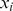 to the output is 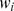.

### Connectivity

**Figure 9:**
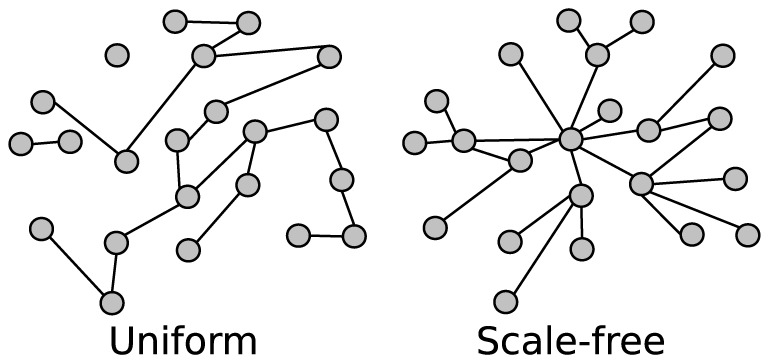
Illustration of two types of connectivity model. A uniform connection policy produces variable length chains of connections with some groups of neurons disconnected from others. A scale-free connection policy leads to a structure of a few highly connected hubs and many sparsely connected leaves.



The type of connectivity used determines the topology of the recurrent network structure in the reservoir. As the synaptic plasticity models used in this work only modify the weights, not the topology, different connectivities will maintain their characteristic structures throughout the simulations. The following connection models are used to probabilistically connect reservoir nodes:

1. **Uniform random:** The probability for any two neurons to be connected is a fixed value 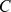. To add a new connection, source and target neurons are both selected randomly with a uniform distribution. This leads to an Erdős-Rényi type network structure [16]. An illustration is provided in Figure 9.
2. **Scale-free:** In a network with the scale-free property, the degree distribution – the number of connections for each node – follows a power law: 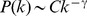 [17]. The probability 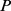 of a node having 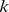 connections, is scaled by some constant 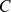. For a growth model when adding new connections, we use the Barabasi-Albert model.

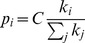
(2)
  This leads to a structure with densely connected hubs. An illustration is provided in Figure 9.
3. **Distance based lattice:** The original model for LSM connectivity [3] arranged neurons in a 3D grid with the probability of a connection between two nodes, inversely proportional to the distance between them. The formula defining the probability of a connection between two neurons is as follows:

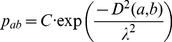
(3)Where 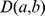 is the Euclidean distance between neurons 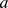 and 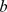. The parameter 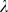 controls both the average number of connections and the average distance with which neurons are connected by.

### Plasticity

**Figure 10:**
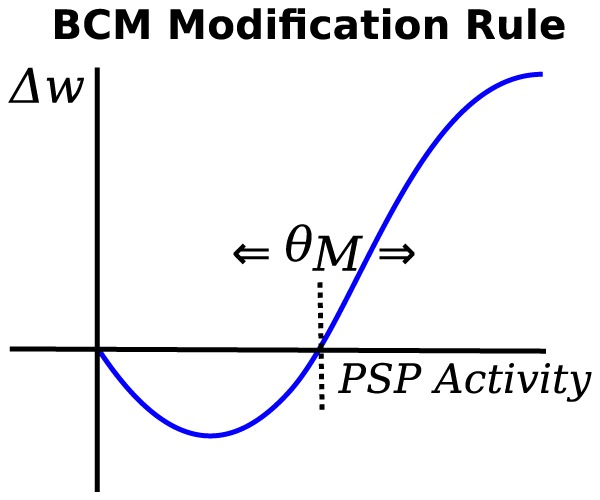
The Bienenstock-Cooper-Munro plasticity rule illustrated with synaptic weight change on the y-scale and post-synaptic activity on the x-scale. 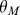
 is the sliding modification threshold that changes based on a temporal average of post-synaptic activity.



**Figure 11:**
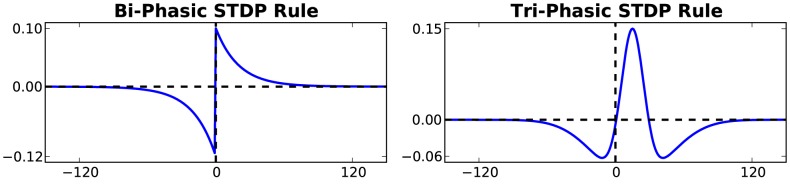
The two predominantly studied STDP learning windows.

Three synaptic plasticity mechanisms are employed in this study, each of them based on the Hebbian postulate [18] of “neurons that fire together, wire together”. The BCM rule regulates the spike rate of the post-synaptic neuron to match a desired rate of spiking. Spike timing dependent plasticity (STDP) is also utilised with two forms of learning window that have been observed in biological experiments. Each mechanism is outlined as follows:

1. **BCM rule:** The BCM rule [19] is a rate based Hebbian rule that also regulates the post-neuron firing rate to a desired level. It works on a temporal average of pre- and post-synaptic activity. The BCM rule is given in Equation 4. The regulating parameter is the dynamic threshold 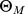, which changes based on the post-synaptic activity 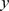 and the desired level 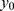 in the following relationship: 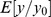, where 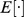 denotes a temporal average. There is also a decay parameter 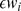 for additional stability, that slowly reduces connection strength and so provides a mechanism for uniform weight decay, irrespective of the level of activity or correlation. A plot of the BCM weight change is presented in Figure 10.
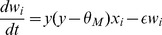
(4)
2. **Bi-phasic STDP:** The STDP rule depends on the temporal correlation between pre- and post-synaptic spikes. The synaptic weight change is computed based on the delay between the firing times of the pre- and post- neuron. This is described in a fixed ‘learning window’ in which the y-axis is the level of weight change and the x-axis is the time delay between a pre- and post-synaptic spike occurrence. The bi-phasic STDP rule consists of two decaying exponential curves [20], a positive one to potentiate in-order spikes, and a negative one to depress out-of-order spikes. This rule was derived from experimental work carried out on populations of neurons *in vitro* [21] [22]. Bi-phasic STDP is given in Equation 5.
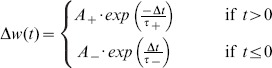
(5)
3. **Tri-phasic STDP:** A tri-phasic STDP learning window consists of a narrow potentiating region for closely correlated activity but depressing regions on either side: for recently uncorrelated activity, and for correlated but late activity. This learning window has been observed *in vitro*, most notably in the hippocampi, between areas CA3 and CA1 [23]. The tri-phasic STDP is given in Equation 6 from [24].

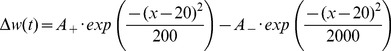
(6)Both STDP learning windows are plotted in Figure 11.

### Reservoir Metrics

#### Class Separation

The class separation is a measure of the comparative distance between the reservoir states corresponding to different classes of stimuli. It was first introduced in [11] and further expanded in [25] as a way to determine how well a reservoir can distinguish one class of inputs from another based on the geometric distance between the class centroids. The reservoir states are taken to define the multi-dimensional coordinates of each sample. Class separation is defined in Equations 8,9,7 as follows:
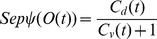
(7)

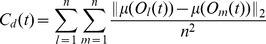
(8)

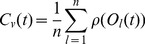
(9)


The class separation 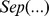, for a given reservoir 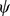 and set of state vectors 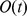, is defined in Equation 7. It is the inter-class distance divided by the intra-class variance, with 1 added to the denominator to prevent dividing by zero. Inter-class distance is calculated according to Equation 8. The class centroids are calculated as the mean state vector for a given class, denoted by 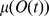. There are 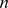 classes in total. Intra-class variance is calculated according to Equation 9. The within-class variance is given as 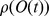. It is calculated by summing the geometric distance between each state vector and its corresponding class average, 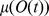.

The rationale behind this class separation measure is that if the distance between different classes of inputs is higher than the distance within the classes, it will be easier for a linear readout to learn a set of weights that distinguishes between the reservoir states of different classes.

#### Kernel Quality

The kernel quality, introduced in [26], is a class-agnostic measure of the reservoir's ability to separate input patterns, in so far as it is independent of the target output. However, it is not quite a task independent measure of a reservoir, due to the dependence of the task-specific input patterns in forming the reservoir states. Like class separation, kernel quality is based on the complete set of 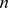 reservoir states produced by input stimuli. Here, a matrix 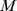 is formed from all of the collected reservoir state vectors, each of which forms one column of dimension 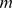. The rank 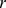 of 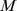 is then taken to be a measure of the computational power of the reservoir, with the maximum rank, and highest computational power to be 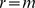, assuming that the number of state vectors is greater than the dimension, 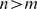. When this is the case, each column in 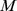 cannot be computed from a linear combination of any other column and therefore it is possible for a linear readout to separate each one of the reservoir states to produce different outputs.

This measure is also referred to as the *linear separation property.*

#### Lyapunov's Exponent

Lyapunov's exponent estimate is a method of calculating the amount of chaos in the dynamics of the reservoir activity. The principle is based on the assumption that internal activity, 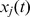, that is generated based on the input signal, 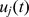, should vary in accordance with that signal, in a system with orderly dynamics. We use the calculation method defined in [27] which was formulated based on theory described in [12]. This method is defined in Equation 10. It is scaled by an undetermined constant 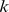 and so can be taken as proportional to the Lyapunov exponent. Therefore it can be compared only to other values using this method, not to other studies, unless the constant 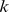 were determined for both.
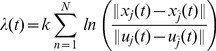
(10)


#### Spectral Radius

The spectral radius [4] is a measure taken directly on the weight matrix of the reservoir, rather than the reservoir states as the others are. It is the largest absolute eigenvalue of the weight matrix that indicates the scale of the weight values. Having a spectral radius less than 1 implies that input driven activity will fade within the network over time. By having a spectral radius exceeding 1, the reservoir dynamics would reach an unstable regime where the activity continually perpetuates and interferes with future inputs. It is therefore suggested that this value be kept below 1, while being a high as possible to allow time-series samples to interact in sufficiently long time-scales. However, the concept of spectral radius assumes that the node activation has a unity output function. It is not clear what implications this metric has with spiking neuron models with connection delays.

### Time Series Tasks

**Figure 12:**
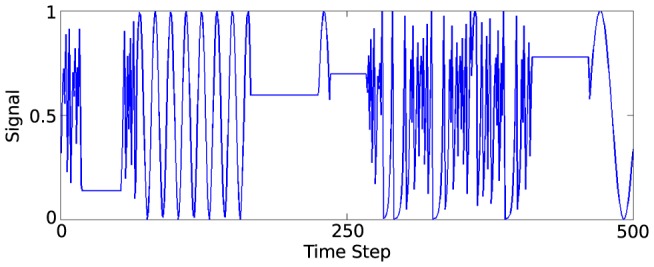
Plot of 500 of the 50,000 data samples generated according to Jaeger's time-series benchmark [28].

1. **Tri-function generator:** A synthetic benchmark is taken from a study performed by Jaeger on ESNs [28]. The task is to predict which of three signal generating functions is currently active in producing a varying input signal. To generate a sample of the signal at a given timestep, one of the three following function types is used; 1) A sine function of a randomly selected period, 2) A chaotic iterated tent map, 3) A randomly chosen constant. The generator is given some low probability, 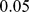, of switching to another function at each time-step. The full method of generating the data is described in [28]. Part of the generated signal is presented in Figure 12.
2. **Speaker recognition:** A speaker recognition task is a classification problem dealing with mapping time-series audio input data to target speaker labels. We use a data set taken from [29] which consists of utterances of 9 male Japanese speakers pronouncing the vowel **/ae/**. The task is to correctly discriminate each speaker based on the speech samples. Each sample is comprised of a sequence of 12 feature audio frames. The features of each frame are the LPC cepstrum coefficients. The sample sequence ranges between 7-29 frames. The dataset is divided into training and testing sets of 270 and 370 samples each, respectively. Note that unlike the benchmark data used in this report, the samples are not in a time-series, yet each sample consists of a time-series of audio frames.
